# Efficacy of repeated PSMA PET-directed radiotherapy for oligorecurrent prostate cancer after initial curative therapy

**DOI:** 10.1007/s00066-020-01629-5

**Published:** 2020-05-12

**Authors:** Christoph Henkenberens, Ann-Kathrin Oehus, Thorsten Derlin, Frank Bengel, Tobias L. Ross, Markus A. Kuczyk, Stefan Janssen, Hans Christiansen, Christoph A. J. von Klot

**Affiliations:** 1 Department of Radiotherapy and Special Oncology, Hannover Medical School, Carl-Neuberg-Str. 1, 30625 Hannover, Germany; 2 Department of Nuclear Medicine, Hannover Medical School, Carl-Neuberg-Str. 1, 30625 Hannover, Germany; 3 Department of Urology and Urologic Oncology, Hannover Medical School, Carl-Neuberg-Str. 1, 30625 Hannover, Germany; 4 Medical practice for Radiotherapy and Radiation Oncology, Treibesstraße 11, 31134 Hildesheim, Germany; 5 Department of Radiation Oncology, University of Lübeck, Ratzeburger Allee 160, 23562 Lübeck, Germany

**Keywords:** PSMA, Biochemical progression, Metastatic Prostate cancer, Oligometastases, Radio-oncology

## Abstract

**Purpose:**

To assess the outcome of prostate cancer (PCa) patients diagnosed with oligorecurrent disease and treated with a first and a second PSMA (prostate-specific membrane antigen ligand) PET(positron-emission tomography)-directed radiotherapy (RT).

**Patients and methods:**

Thirty-two patients with oligorecurrent relapse after curative therapy received a first PSMA PET-directed RT of all metastases. After biochemical progression, all patients received a second PSMA PET-directed RT of all metastases. The main outcome parameters were biochemical progression-free survival (bPFS) and androgen deprivation therapy-free survival (ADT-FS). The intervals of BPFS were analyzed separately as follows: the interval from the last day of PSMA PET-directed RT to the first biochemical progression was defined as bPFS_1 and the interval from second PSMA PET-directed RT to further biochemical progression was defined as bPFS_2.

**Results:**

The median follow-up duration was 39.5 months (18–60). One out of 32 (3.1%) patients died after 47 months of progressive metastatic prostate cancer (mPCa). All patients showed biochemical responses after the first PSMA PET-directed RT and the median prostate-specific antigen (PSA) level before RT was 1.70 ng/mL (0.2–3.8), which decreased significantly to a median PSA nadir level of 0.39 ng/mL (range <0.07–3.8; *p* = 0.004). The median PSA level at biochemical progression after the first PSMA PET-directed RT was 2.9 ng/mL (range 0.12–12.80; *p* = 0.24). Furthermore, the PSA level after the second PSMA PET-directed RT at the last follow-up (0.52 ng/mL, range <0.07–154.0) was not significantly different (*p* = 0.36) from the median PSA level (1.70 ng/mL, range 0.2–3.8) before the first PSMA PET-directed RT. The median bPFS_1 was 16.0 months after the first PSMA PET-directed RT (95% CI 11.9–19.2) and the median bPFS_2 was significantly shorter at 8.0 months (95% CI 6.3–17.7) after the second PSMA PET-directed RT (*p* = 0.03; 95% CI 1.9–8.3). Multivariate analysis revealed no significant parameter for bPFS_1, whereas extrapelvic disease was the only significant parameter (*p* = 0.02, OR 2.3; 95% CI 0.81-4.19) in multivariate analysis for bPFS_2. The median ADT-FS was 31.0 months (95% CI 20.1–41.8) and multivariate analysis showed that patients with bone metastases, compared to patients with only lymph node metastases at first PSMA PET-directed RT, had a significantly higher chance (*p* = 0.007, OR 4.51; 95% CI 1.8–13.47) of needing ADT at the last follow-up visit.

**Conclusion:**

If patients are followed up closely, including PSMA PET scans, a second PSMA PET-directed RT represents a viable treatment option for well-informed and well-selected patients.

## Introduction

The cornerstone of treatment for non-castrated metastatic prostate cancer (mPCa) is androgen deprivation therapy (ADT), which has remained unchanged over the past years [1]. The negative impact of ADT on quality of life (QoL) [1] has resulted in a search for alternatives for well-selected patients using personalized treatment concepts on the basis of prostate-specific membrane antigen ligand positron-emission tomography (PSMA PET) [2–5]. There is increasing evidence that patients with a limited number of metastases have a better prognosis than patients with widespread metastatic disease, and data outside large prospective trials suggest that metastasis-directed therapies (MDTs) for mPCa patients with a so-called “oligometastatic status,” based on a generally accepted imaging-based cut-off of five metastases, improve the clinical outcome [2, 6]. The recent introduction of PSMA ligand PET has substantially improved the diagnostic accuracy of staging at low prostate-specific antigen (PSA) levels [7–11], allowing refined and well-monitored individualized radio-oncological treatment concepts that aim to improve PSA kinetics, prolong progression-free survival, defer the initiation of ADT, and potentially cure the patient [2–5, 12]. The STOMP [13] and POPSTAR trials [14], as well as the data published by Kneebone et al. [15], demonstrated that MDT alone might delay ADT for a relevant period. However, approximately half of the patients will develop oligoprogressive disease after MDT [13], making these patients amenable to repeated MDT and further improving the PSA kinetics and delaying the initiation of ADT [16]. Data on the feasibility and clinical outcome of a second MDT guided by PSMA PET imaging after previous PSMA PET-directed radiotherapy (RT) are very limited.

Thus, in the current study, we assessed the outcomes of patients with mPCa diagnosed with oligorecurrent disease after initial curative therapy who were treated with a second PSMA PET-directed radiotherapy (RT).

## Patients and methods

Between June 2014 and February 2019, 32 hormone-naïve patients with oligorecurrent PCa after primary curative therapy received a first PSMA PET-directed RT as MDT for all metastases, and after oligoprogression, the patients received a definitive second PSMA PET-directed RT for all metastases. This retrospective study was approved by the local ethics committee (no. 3661-2017), and all cases were discussed and approved for RT by the multidisciplinary uro-oncologic board. Oligometastatic disease was defined as ≤5 visceral, bone, or lymph node metastases. The patients’ characteristics are summarized in Table 1.

**Table 1:** Patient characteristics

Characteristics	Median (range); *n* (%)
*Age at PCa diagnosis (years)*	63 (53–74)
*Initial PSA (ng/ml)*	9.8 (3.7–47.4)
*Primary therapy*	
RPE alone	11 (34.4)
RPE + aRT	9 (28.1)
RPE + sRT	10 (31.2)
EBRT + temporary ADT	2 (6.3)
cT1c	2 (6.3)
pT2c	13 (40.6)
pT3a	4 (12.5)
pT3b	12 (37.5)
pT4	1 (3.1)
*Initial N stage*	
N0	28 (87.5)
N1	4 (12.5)
*Surgical margins*	
R0	28 (93.3)
R1	2 (6.7)
*Initial risk group*	
High	19 (59.4)
Very high	13 (40.6)
*Initial PSA (ng/ml)*	9.8 (3.7–47.4)
*PSA nadir after definitive therapy (ng/ml)*	0.07 (<0.07–3.0)
*Interval (months) from definitive therapy to PSMA PET*	47 (12–168)
*PSA level at PSMA-PET imaging (ng/ml)*	1.70 (0.2–4.8)
*Patients with ADT at PSMA-ligand PET imaging*	0 (%)
*Median PSA-dt at time of PSMA-PET imaging (months)*	8.7 (3.1–17.2)
<6	7 (21.9)
>6, <12	10 (31.2)
>12	15 (46.9)

*ADT* androgen deprivation therapy, *aRT* adjuvant radiotherapy, *dt* doubling time, *EBRT* external beam radiation therapy, *PCa* prostate cancer, *PSMA-PET* prostate-specific membrane antigen positron-emission tomography, *PSA* prostate-specific antigen, *RP* radical prostatectomy, *sRT* salvage radiotherapy

### PET imaging

Each patient received PET imaging with a ^68^gallium-labeled PSMA ligand [17] and imaging was performed according to the joint EANM and SNMMI guidelines [18]. PSMA PET scans were acquired in conjunction with low-dose computed tomography (CT) on a dedicated PET/CT system (Siemens Biograph mCT 128 Flow; Siemens, Knoxville, TN, USA) equipped with an extended field-of-view lutetium oxyorthosilicate PET component, a 128-slice spiral CT component, and a magnetically powered table optimized for continuous scanning. No intravenous contrast material was administered. All patients gave written informed consent before PSMA PET/CT. A positive visual assessment of increased focal tracer uptake higher than the surrounding background activity was used as the criterion for malignancy [9].

### Radiotherapy treatment

Patients with lymph node metastases or relapse in the prostatic fossa were treated with conventionally fractionated RT (CF-RT) and patients with bone metastases were treated with slightly hypofractionated RT (HF-RT). In the case of lymph node metastases, the clinical target volume (CTV) encompassed the lymph drainage vessel to the next bifurcation without the whole ipsilateral lymphatic drainage vessel; the dose was 50.0 Gy (single dose 2.0 Gy), followed by a sequential CF-RT boost of 10.0 Gy (single dose 2.0 Gy) to the lymph node metastases. Relapse in the prostate bed was treated with CF-RT doses of 70.0–74.0 Gy (single dose of 2.0 Gy). Bone metastases were treated with HF-RT and a single dose of 2.5 Gy up to a dose of 45.0 Gy. The planning target volume (PTV) for lymph node metastases, bone metastases, and local relapse in the prostatic fossa included the CTV plus a 10-mm safety margin in all directions to account for setup errors. Image guidance was conducted at least twice a week with megavoltage cone-beam CT. Visceral metastases were treated with image-guided stereotactic body radiation therapy (SBRT) and a total dose of 37.5 Gy (single dose 12.5 Gy) to the encompassing 67% PTV isodose. The PTV included the internal target volume (ITV) plus a 4-mm safety margin in all directions to account for setup errors.

### Follow-up and endpoints

All patients had periodic urologic follow-up evaluations, which included PSA measurements every 3 months. Biochemically progressive disease after RT was defined as two consecutive increases in PSA levels from the nadir PSA level or a PSA level above baseline. Biochemical nonresponse was defined as the first PSA level 3 months after RT that was ≥10% of the baseline PSA level at the time of PSMA PET/CT scan before RT. Patients who met the criteria of biochemical progression had a second PSMA PET/CT for restaging purposes, which was used for the second PSMA PET-directed RT to irradiate all new metastases. Patients with extensive disease (≥6 metastases) received systemic therapy according to the urologist’s choice. In general, outcomes were defined from the last day of RT. The outcome parameters were biochemical progression-free survival (bPFS), ADT-free survival (ADT-FS), overall survival (OS), and toxicity. The intervals of BPFS were analyzed separately as follows: the interval from the last day of first PSMA PET-directed RT to the first biochemical progression was defined as bPFS_1 and the interval from second PSMA PET-directed RT to further biochemical progression was defined as bPFS_2. RT-associated toxicity was analyzed using the National Cancer Institute Common Terminology Criteria for Adverse Events (CTCAE) v4.0 [19]. To assess the local failure rate we performed a coregistration of the PSMA PET/CT scans with the RT treatment plans. Focally increased tracer uptake higher than the surrounding background within the PTV was classified as infield relapse.

### Statistical analysis

For statistical analysis, SPSS Statistics v25.0 (IBM, Armonk, New York, USA) was used. We used the paired Student’s *t*-test to compare pre-RT with post-RT parametric parameters and the Wilcoxon signed-rank test when the data were not normally distributed. The time-to-event data were calculated using the Kaplan–Meier method. Factors for RT treatment failure were analyzed with the log rank test in univariate analyses and significant factors were further assessed with multivariate analyses to identify independent variables for bRFS and ADT-FS. *P*-values <0.05 were considered statistically significant. Graphical presentations of the patterns of progression were created using a free software for statistical computing and graphics (R version 3.0.3).

## Results

### PSMA PET staging and therapy for metastases

A total of 59 PSMA ligand-positive metastases were detected and treated with RT. Of these, 66.1% (39/59) were pelvic lymph node metastases, 5.1% (3/59) were periaortic lymph node metastases, 22.0% (13/59) were bone metastases, 1.7% (1/59) were visceral metastases, and 5.1% (3/59) were local relapses in the prostatic fossa. A total of 53.1% of patients (17/32) had only lymph node metastases, 31.3% (10/32) of patients had only bone metastases, 12.5% (4/32) of patients had lymph node and bone metastases, and 2.6% (1/32) of patients had visceral metastases.

### Patterns of progression and patient outcomes

Table 2 summarizes the results of PSMA PET scans and first PSMA PET-directed RT, as well as the results from PSMA PET restaging after biochemical progression and a consecutive second PSMA PET-directed RT. Analysis of the RT treatment plans and the PSMA PET/CT scans for all metastases at first (*n* = 59) and second (*n* = 60) PSMA PET-directed RT resulted in a cumulative infield relapse rate of 3.4% (4/119) and in a cumulative outfield relapse rate of 96.6% (115/119). The four infield relapses occurred in the right iliac lymph nodes (25%, 1/4), in a rib metastasis (25%, 1/4), and in pelvic bone metastases (50%, 2/4).

**Table 2:** Results of PSMA ligand PET staging prior to PSMA ligand-based radiotherapy and PSMA ligand PET restaging prior to second PSMA ligand-based radiotherapy

	PSMA ligand-based RT	Second PSMA ligand-based RT
	*N (%)*	*N (%), p-value*
*No. of PSMA ligand-positive lesions*	59 (100)	60 (100); 0.90
Total no. of LNs	42 (69.2)	36 (60); 0.51
…Pelvic LNs	39 (36.2)	15 (25); 0.03
…Periaortic/interaortocaval LNs	3 (27.6)	19 (31.7); 0.09
Total no. of bone metastases	13 (24.3)	23 (38.3); 0.04
…Pelvic bone	7 (13.0)	10 (16.7); 0.37
…Spinal bone	4 (5.4)	11 (18.3); 0.14
…Other	2 (5.9)	2 (3.3); 0.64
Prostatic fossa	3 (6.5)	0 (0); 0.32
Total no. of visceral metastases	1 (1.7)	1 (1.7); 0.91
*Concurrent ADT at radiotherapy*	0 (0.0)	3 (9.4); 0.32
*Other local therapies (e.g., surgery, RFA)*	0 (0.0)	0 (0.0)
	*Median (range)*	*Median (range); p-value*
*PSA before RT*	1.71 (0.21–3.8)	2.90 (0.12–22.80); 0.23
*PSA-dt (months)*	8.7 (3.1–17.2)	5.8 (1.6–8.2); 0.05
*No. irradiated metastases*	2 (1–4)	2 (1–5); 0.90

*ADT* androgen deprivation therapy, *dt* doubling-time, *LNs* lymph node metastases, *PCa* prostate cancer, *PSMA-PET* prostate-specific membrane antigen positron-emission tomography, *PSA* prostate-specific antigen, *RFA* radiofrequency ablation, *sRT* salvage radiotherapy, *SBRT* stereotactic body radiation therapy, *SIB* simultaneous integrated boost

**Fig. 1:**
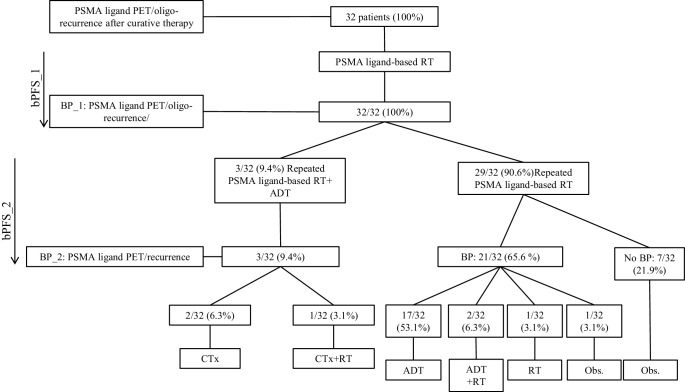
Schematic overview of the treatment and relapse patterns of oligorecurrent prostate cancer after first and second PSMA PET-directed RT. *ADT* androgen deprivation therapy, *BP* biochemical progression, *BP_1* first biochemical progression, *BP_2* second biochemical progression, *bPFS_1* interval from last day of PSMA PET-directed RT to first biochemical progression, *bPFS_2* interval from second PSMA PET-directed RT to further/second biochemical progression, *CT* chemotherapy, *Obs* observation, *PET* positron-emission tomography, *PSMA* prostate-specific membrane protein, *RT* radiotherapy

**Fig. 2:**
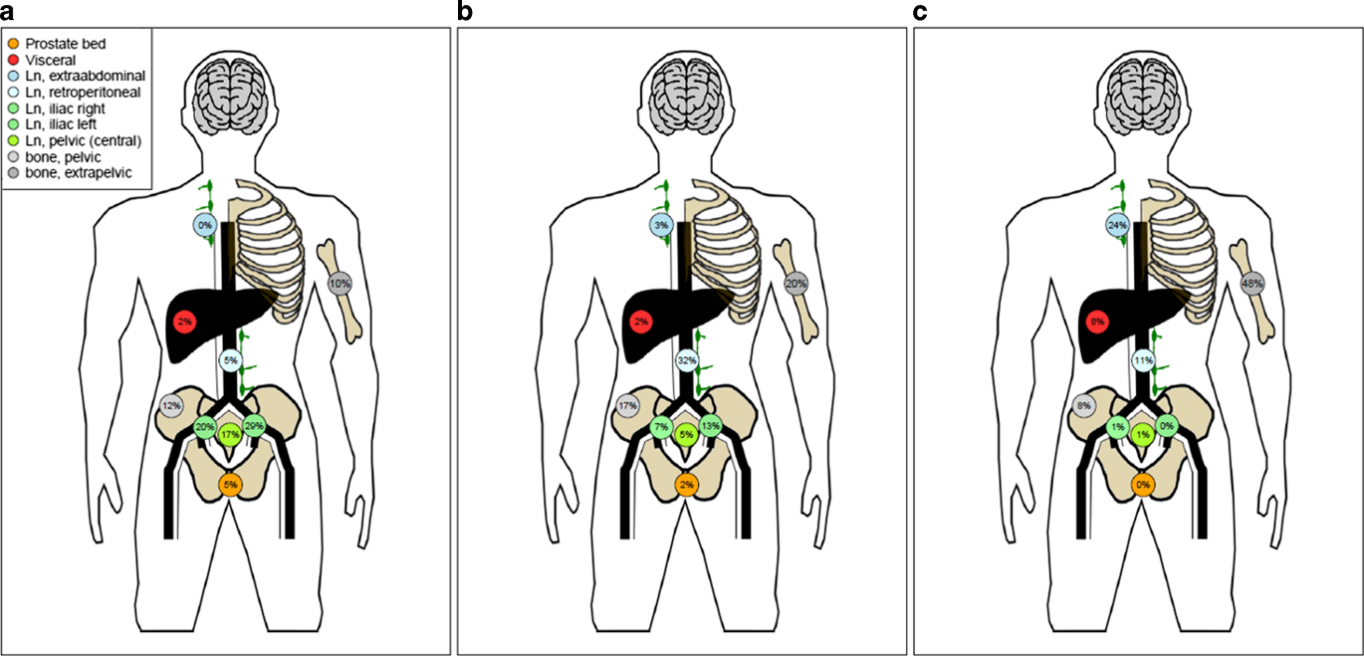
Schematic illustration of the distribution of metastases at oligorecurrence based on PSMA PET/CT after curative initial therapy for prostate cancer (**a**), distribution of metastases at oligoprogression after first PSMA PET-directed RT (**b**) and distribution of metastases at further progression after second PSMA PET-directed RT (**c**) showing local control in the pelvis and the increase in extrapelvic lymph node and extrapelvic bone metastases. *Ln* lymph nodes

**Fig. 3:**
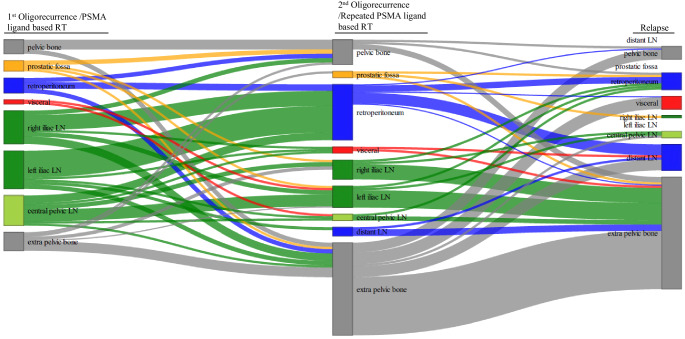
Sankey plot showing the location of new metastases in relation to prior metastatic sites (for every patient, every metastatic lesion was counted). On the left side, the initial distribution at oligorecurrence at the time of first PSMA PET-directed RT is shown; in the middle, the distribution of metastases at the time of second PSMA PET-directed RT is shown; and on the right side, the distribution after second PSMA ligand-directed RT is shown. Similar to the analysis of the involved anatomic regions (Fig. 2), we observed a numerical shift from iliac lymph node metastases to retroperitoneal lymph node metastases and marked skeletal involvement, particularly involving the extrapelvic skeletal system. *PSMA* prostate-specific membrane antigen, *RT* radiotherapy, *LN* lymph node

The median follow-up time was 39.5 months (18–60). One out of 32 (3.1%) patients died after 47 months of progressive mPCa. All patients showed biochemical responses after the first PSMA PET-directed RT: the median PSA level before first RT was 1.70 ng/mL (0.2–3.8), which decreased significantly to a median PSA nadir level of 0.39 ng/mL (range <0.07–3.8; *p* = 0.004). The median PSA level at biochemical progression after the first PSMA PET-directed RT was 2.9 ng/mL (range 0.12–12.80; *p* = 0.24). Furthermore, the PSA level after the second PSMA PET-directed RT at the last follow-up (0.52 ng/mL, range <0.07–154.0) was not significantly different (*p* = 0.36) from the median PSA level (1.70 ng/mL, range 0.2–3.8) before the first PSMA PET-directed RT. Additionally, 12.5% (4/32) of patients did not show a biochemical response after second the PSMA PET-directed RT and were classified as nonresponders. Fig. 1 gives a schematic overview of first and second progressions following the first and second PSMA PET-directed RT, respectively. Figs. 2 and 3 show the initial distribution of metastases at the time of the first PSMA PET-directed RT and the location of new metastases at the time of the second PSMA PET-directed RT, as well as at the time of further progression after the second PSMA PET-directed RT.

**Fig. 4:**
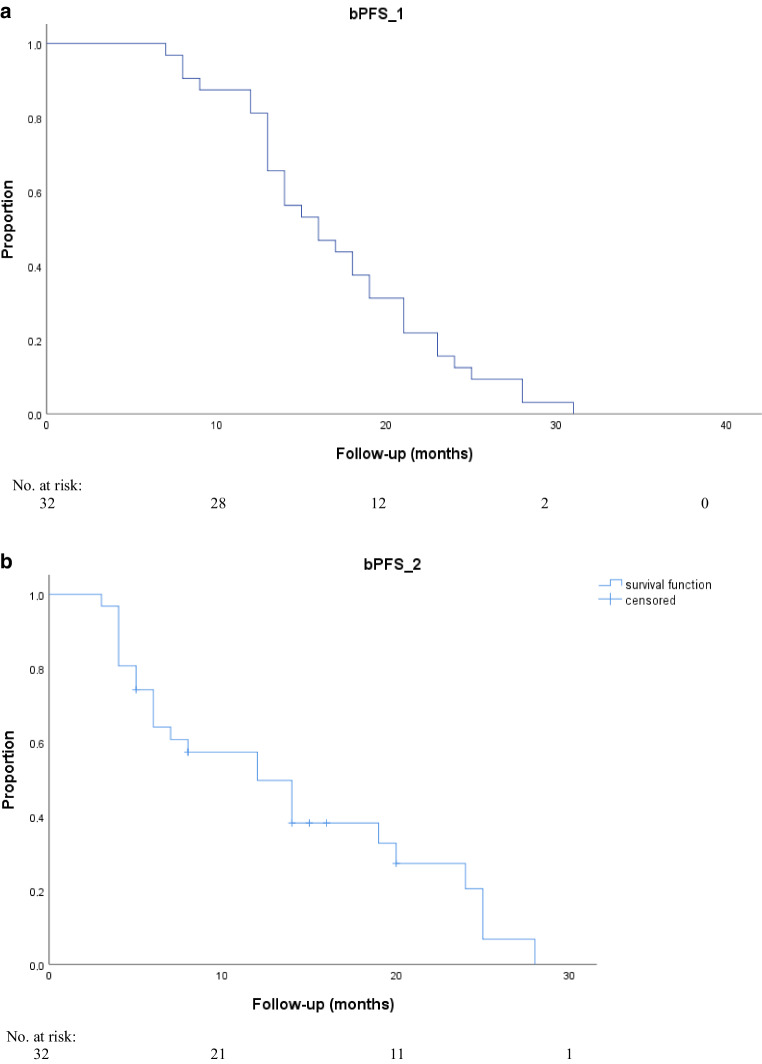
Kaplan–Meier curves of biochemical progression-free survival (bPFS) after first PSMA PET-directed radiotherapy (*bPFS_1*; **a**) and after second PSMA PET-directed radiotherapy (*bPFS_2*; **b**)

The median bPFS_1 was 16.0 months after first PSMA PET-directed RT (95% CI 11.9–19.2; Fig. 4a) and the median bPFS_2 was significantly shorter at 8.0 months (95% CI 6.3–17.7; Fig. 4b) after second PSMA PET-directed RT (*p* = 0.03; 95% CI 1.9–8.3). None of the parameters significant for bPFS_1 in the univariate analyses reached significance in multivariate analyses. Extrapelvic disease was the only significant parameter (*p* = 0.02, OR 2.3; 95% CI 0.81–4.19) in multivariate analysis for bPFS_2. Table 3 shows the detailed results of uni- and multivariate analyses for bPFS_1 and bPFS_2.

**Table 3:** Results of uni- and multivariate analyses for first and second biochemical progression-free survival (bPFS_1, bPFS_2)

	bPFS_1		bPFS_2	
	Univariate analysis	Multivariable analysis	Univariate analysis	Multivariable analysis
	*p*-value	*p*-value, OR (95% CI)	*p*-value	*p*-value, OR (95% CI)
Initial T stage (≤T2 vs. ≥T3)	0.91	–	0.96	–
Initial N stage (N0 vs. N1)	0.39	–	0.67	–
Initial PSA level in ng/ml (≤20 vs. >20)	0.81	–	0.82	–
Initial PSA level in ng/ml (≤10 vs. >10)	0.92	–	0.54	–
PSA nadir after RP (≤0.07 ng/mL vs. >0.07 ng/mL)	0.33	–	0.39	–
Gleason score (≤7 vs. ≥8)	0.18	–	0.22	–
No. of removed LN at RP (≤15 vs. >15)	0.63	–	0.34	–
Initial risk group (high risk vs. very high risk)	0.32	–	0.54	–
PSA-dt (months; ≤6, >6)	0.02	0.12, 1.92 (1.02–8.52)	0.19	–
PSA dt (months, ≤12, >12)	0.05	0.23, 1.76 (0.34–8.71)	0.46	–
No. of irradiated metastases (1 vs. >1)	0.73	–	0.19	–
No. of irradiated metastases (≤2 vs. ≥3)	0.23	–	0.05	0.56, 0.73 (0.29–1.28)
No. of irradiated metastases (≤3 vs. ≥4)	0.24	–	0.20	–
Type of metastases (LNs vs. bone)	0.45	–	0.04	0.19, 0.64 (0.23–1.39)
Extrapelvic disease (LNs and/or bone)	0.92	–	0.02	0.02, 2.3 (0.81–4.19)

*dt* doubling time, *LN* lymph node, *PSA* prostate-specific antigen, *RP* radical prostatectomy

**Fig. 5:**
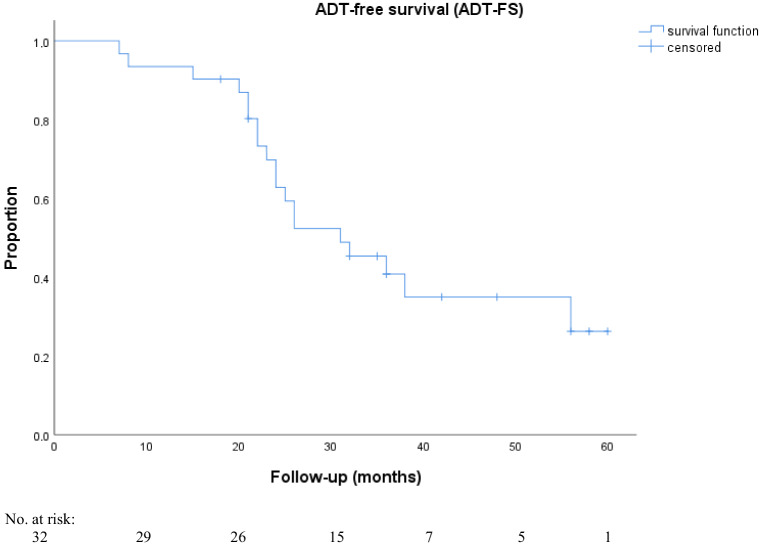
Kaplan–Meier curves of androgen deprivation therapy-free survival (*ADT-FS*) after first and second PSMA ligand PET-directed radiotherapy

At the last follow-up, 28.1% (9/32) of patients did not need ADT. The median ADT-FS was 31.0 months (95% CI 20.1–41.8; Fig. 5) and multivariate analysis showed that patients with bone metastases, compared to patients with only lymph node metastases at the first PSMA PET-directed RT, had a significantly higher chance (*p* = 0.007, OR 4.51; 95% CI 1.8–13.47) of needing ADT at the last follow-up visit. Furthermore, patients with oligoprogression outside the pelvis, compared to patients with oligoprogression limited to the pelvis at the second PSMA PET-directed RT, also had a significantly higher risk of needing ADT at the last follow-up visit (*p* = 0.03, OR 3.2; 95% CI 1.2–15.10). Table 4 shows the detailed results of uni- and multivariate analyses for ADT-FS.

**Table 4:** Results of uni- and multivariate analyses for androgen deprivation therapy-free survival (ADT-FS)

	Univariate analysis	Multivariate analysis	
	*p*-value	*p*-value	OR, (95% CI)
Initial T stage (≤T2 vs. ≥T3)	0.19	–	–
Initial N stage (N0 vs. N1)	0.66	–	–
Initial PSA level in ng/ml (≤10 vs. >10)	0.47	–	–
Initial PSA level in ng/ml (≤20 vs. >20)	0.50	–	–
PSA nadir after RP (≤0.07 ng/mL vs. >0.07 ng/mL)	0.97	–	–
Gleason score (≤7 vs. ≥8)	0.10	–	–
No. of removed LNs at RP (≤15 vs. >15)	0.36	–	–
Initial risk group (high risk vs. very high risk)	0.75	–	–
PSA-dt (months; ≤6, >6) at first PSMA ligand-based RT	0.01	0.74	1.25, 0.34–4.60
PSA-dt (months; ≤12, >12) at first PSMA ligand-based RT	0.13	–	–
PSA-dt (months; ≤6, >6) at second PSMA ligand-based RT	0.27	–	–
PSA-dt (months; ≤12, >12) at second PSMA ligand-based RT	0.87	–	–
No. of irradiated metastases (1 vs. >1) at first PSMA ligand-based RT	0.81	–	–
No. of irradiated metastases (no. of irradiated metastases [≤2 vs. ≥3] at first PSMA ligand-based RT)	0.27	–	–
No. of irradiated metastases (no. of irradiated metastases [≤3 vs. ≥4] at first PSMA ligand-based RT)	0.05	0.59	1.68, 0.35–6.02
No. of irradiated metastases (1 vs. >1) at second PSMA ligand-based RT	0.52	–	–
No. of irradiated metastases (≤2 vs. ≥3) at second PSMA ligand-based RT	0.92	–	–
No. of irradiated metastases (≤3 vs. ≥4) at second PSMA ligand-based RT	0.36	–	–
Type of metastases at first PSMA ligand-based RT (lymph node vs. bone)	<0.001	0.007	4.51, 1.8–13.47
Extrapelvic disease at first PSMA ligand-based RT (lymph node vs. bone)	0.31	–	–
Type of metastases at second PSMA ligand-based RT (LN vs. bone)	0.82	–	–
Extrapelvic disease at second PSMA ligand-based RT (LN and/or bone)	0.01	0.03	3.2, 1.2–15.10

*dt* doubling time, *LN* lymph node, *PSA* prostate-specific antigen, *PSMA* prostate-specific membrane antigen, *RP* radical prostatectomy

### Toxicity

Acute grade III toxicity was not observed; 3.1% (1/32) of patients developed grade II acute genitourinary toxicity. Grade II acute gastrointestinal toxicity occurred in 12.5% (4/32) of patients and grade I acute gastrointestinal toxicity occurred in 9.4% (3/32) of patients. Late grade III gastrointestinal toxicity occurred in 3.1% (1/32) of patients and grade II genitourinary toxicity occurred in 3.1% (1/78) of patients.

## Discussion

The implementation of PSMA ligand PET imaging has substantially improved the diagnostic accuracy of detecting metastatic PCa at low PSA levels [7, 8, 20]. Although large randomized prospective studies are lacking, MDT is considered a viable treatment option for well-selected patients with oligorecurrent PCa [21]. Furthermore, there is still controversy about the optimal timing to initiate palliative ADT for asymptomatic metastatic patients because of the lack of prospective trials in the PSA era [1]. Furthermore, ADT offers no curative potential [1] and significantly impairs QoL in a relevant number of patients [22].

Smaller prospective trials with heterogeneous patient cohorts, including one with choline PET imaging [13], one with PSMA ligand PET imaging [14], and one with sodium fluoride (NA-F) PET imaging [15], showed encouraging results for MDT in oligometastatic prostate cancer. The STOMP [13] and POPSTAR trials [14], as well as the data published by Kneebone et al. [15], demonstrated that MDT alone might delay ADT for a relevant period. However, patients with MDT alone develop biochemical progression earlier than patients with MDT plus ADT [13–15]. To the best of the authors’ knowledge, there are no published data comparing MDT alone versus MDT plus ADT with regard to hard endpoints such as prostate carcinoma-specific survival. In our opinion, patients must be well informed that ADT is the standard of care and any type of MDT is an individual treatment concept outside the current guidelines, although the optimal timing of initiation of ADT at low PSA levels remains unknown [1]. Nevertheless, approximately half of the patients will develop oligoprogressive disease after MDT alone [7], making these patients amenable to a second MDT and further delaying the start of ADT [13, 23, 24]. Additionally, due to the lack of data from prospective trials, the current guidelines do not reflect the diagnostic accuracy of PSMA ligand PET staging at low PSA levels to select patients who should or should not receive ADT [1].

To the best of our knowledge, a second RT for oligorecurrent PCa on the basis of PSMA ligand PET staging and restaging has never been reported. We showed that 28.1% (9/32) of patients did not need ADT at the last follow-up, resulting in a median ADT-FS of 31.0 (95% CI 20.1–41.8) months, which is approximately the same as the duration that other authors have reported [13, 23, 24]. None of the published data [13, 23, 24] included PSMA ligand PET staging for MDT that led to MDT at higher PSA levels, particularly increasing the likelihood that patients had an underestimated extent of lymph node metastases [11, 22]. In addition, the initiation of ADT based on the urologist’s choice at low PSA levels might be a confounder for ADT-FS and, therefore, hamper the comparison of these retrospective data.

We found that the type of irradiated metastases at oligorecurrence was the most important clinical parameter for ADT-FS. Patients with bone metastases had a significantly higher risk (*p* = 0.007, OR 4.51; 95% CI 1.8–13.47) of needing ADT at their last follow-up compared to patients with only lymph node metastases. Population-based data supported our findings because patients with bone metastases have a worse prognosis and lower cancer-specific survival (CSS) than those with lymph node metastases [25, 26]. A recently published SEER database analysis suggested that patients with stage M1a tumors receive significantly greater clinical benefits from local therapies to the prostate than patients with stage M1b tumors [27]. Although the median number of irradiated metastases per patient at the first PSMA PET-directed RT and at the second PSMA PET-directed RT was the same (*n* = 2; *p* = 0.90), the median BPFS_2 after the second PSMA PET-directed RT was significantly shorter than the median BPFS_1 after the first PSMA PE-directed RT (8.0 months vs. 16.0 months, *p* = 0.03; 95% CI 1.9–8.3). Moreover, the shorter BPFS after the second PSMA PET-directed RT was associated with a more widespread pattern of metastases (increase in bone metastases and extrapelvic lymph node metastases), which is a possible indicator for an evolving tumor biology. Until now, the evolutionary history of metastatic prostate cancer of monoclonal versus polyclonal cell seeding leading to a linear versus branching pattern of metastatic spread [28] at low PSA levels remains unknown. Many genomic and nongenomic biomarkers have been investigated in mPC [29, 30], but none of these markers are available outside of dedicated study protocols for clinical routine. On the other hand, exploratory analyses of patients with limited tumor burden according to the CHAARTED criteria revealed no OS benefit for escalated systemic therapy using either the combination of ADT + docetaxel [31] or ADT + enzalutamide [32] compared to ADT alone, indicating a different biology. With regard to PSA kinetics as a biomarker, we found that the median PSA-dt at the second PSMA PET-directed RT was significantly shorter than the median PSA-dt at the first PSMA PET-directed RT (5.8 versus 8.7 months, *p* = 0.05), indicating a biologically more aggressive disease at oligoprogression after the first PSMA PET-directed RT. Furthermore, a PSA-dt <6 months, compared to a PSA-dt >6 months, showed a trend towards significance (*p* = 0.12) in multivariate analyses for a worse BPFS_1. Ost el. showed that patients with non-castrate mPCA and one metastasis plus a PSA-dt >3 months had a 5-year CSS rate of 100%, whereas patients with a PSA-dt <3 months plus >1 metastasis had a 5-year CSS rate of only 8% [33]. There is controversy about the radiation dose, field size, and elective node irradiation when PSMA ligand PET is used for MDT of oligorecurrent mPCa. Data from the choline PET era confirmed that choline PET underestimated the extent of lymph node metastases [34], which is reflected by the fact that approximately two out of three patients treated with SBRT for pelvic lymph node metastases relapsed with lymph node metastases [25, 35], leading to a higher relapse rate than that after elective node irradiation (ENI), although the relapse rate concerning bone and visceral metastases seems to be comparable between SBRT and ENI [36]. The extent of lymph node metastases can be assessed more precisely by PSMA PET than by choline PET [7]. Therefore, we do not recommend ENI because the initiation of systemic therapies according to high- and low-burden disease and the corresponding prognosis do not depend on lymph node metastases, but on bone and visceral metastases [1]. In addition, other retrospective data from the PSMA PET era assessing the pattern of progression of lesion-directed RT without ENI of contralateral lymphatic drainage showed a significant increase in retroperitoneal and osseous metastases and only a very small number of contralateral pelvic recurrences [2]. These results may indicate that ENI may have been a necessary compensator for the poor detection rate of lymph node metastases of choline PET scans.

Some limitations of this study should be acknowledged. The retrospective nature has inherent limitations and might have introduced selection bias, although the presented cohort had a strict follow-up schedule and staging was performed only with PSMA ligand PET. Therefore, fewer metastases should be missed with this method than with conventional imaging and choline PET [11, 20, 35], leading to well-selected patients [37]. Moreover, the second PSMA ligand PET for restaging purposes allows assessment of the metabolic response after RT, leading to a reliable discrimination of new metastases and successfully irradiated metastases [38]. In addition, the study included a selected cohort with only high-risk and very high-risk patients. Therefore, caution should be taken when generalizing the observed results for patients with intermediate- or low-risk oligorecurrent PCa. Additionally, the sample size of 32 patients limited the statistical power, although the observed clinical results are robust and contribute significantly to the discussion of a second PSMA PET-directed MDT after curative primary therapy in a quickly changing clinical field.

## Conclusion

Repeated PMSA PET-directed RT for oligorecurrent prostate cancer postponed ADT without significant toxicities. If patients are followed up closely, including PSMA PET scans, repeated PSMA PET-directed RT represents a viable treatment option for well-informed and well-selected patients.
